# Cities, from Information to Interaction

**DOI:** 10.3390/e20110834

**Published:** 2018-10-31

**Authors:** Vinicius M. Netto, Edgardo Brigatti, João Meirelles, Fabiano L. Ribeiro, Bruno Pace, Caio Cacholas, Patricia Sanches

**Affiliations:** 1 Department of Urbanism, Universidade Federal Fluminense, Rua Passo da Patria 156, Niteroi, Rio de Janeiro 24210-240, Brazil; 2 Instituto de Física, Universidade Federal do Rio de Janeiro (UFRJ), Av. Athos da Silveira Ramos 149, Rio de Janeiro 21941-972, Brazil; 3 Laboratory on Human-Environment Relations in Urban Systems, Ecole Polytechnique Federale de Lausanne HERUS, EPFL, CH-1015 Lausanne, Switzerland; 4 Deparment of Physics (DFI), Universidade Federal de Lavras (UFLA), Caixa postal 3037, Lavras, Brazil; 5 Adalbertstraße 66, PLZ 10179 Berlin, Germany; 6 Programme of Graduate Studies, Universidade Federal Fluminense, Rua Passo da Patria 156, Niteroi, Rio de Janeiro 24210-240, Brazil; 7 Department of Forest Science, Universidade de São Paulo, Avenida Pádua Dias 11, Piracicaba, São Paulo 13418-900, Brazil

**Keywords:** information, cities, interaction, environmental information, entropy, scale, enaction

## Abstract

From physics to the social sciences, information is now seen as a fundamental component of reality. However, a form of information seems still underestimated, perhaps precisely because it is so pervasive that we take it for granted: the information encoded in the very environment we live in. We still do not fully understand how information takes the form of cities, and how our minds deal with it in order to learn about the world, make daily decisions, and take part in the complex system of interactions we create as we live together. This paper addresses three related problems that need to be solved if we are to understand the role of environmental information: (1) the *physical* problem: how can we preserve information in the built environment? (2) The *semantic* problem: how do we make environmental information meaningful? and (3) the *pragmatic* problem: how do we use environmental information in our daily lives? Attempting to devise a solution to these problems, we introduce a three-layered model of information in cities, namely environmental information in physical space, environmental information in semantic space, and the information enacted by interacting agents. We propose forms of estimating entropy in these different layers, and apply these measures to emblematic urban cases and simulated scenarios. Our results suggest that ordered spatial structures and diverse land use patterns encode information, and that aspects of physical and semantic information affect coordination in interaction systems.

## 1. Complex Systems in Relation: Minds, Cities and Societies

Look outside your window. You will see differences in shapes and sizes between buildings, perhaps some taller and more concentrated in certain parts of the city. You will see that these buildings are connected to streets, and that some of these streets are also likely to be different from one another. Even if you have never been in this city or area before, you can walk around and find someone or something you need in a busy street a couple of corners away. You can find your way around it. When you choose a place, you join a situation that culminates networks of previous interactions that you never thought about, but were a condition for you to be there at that moment. All of these situations are of course part of a larger structure. In fact, you are living within a pattern—the interplay of recognizable relations and surprising variations, of hierarchy and contingency. As these patterns involve tangible spaces, social activities and possibilities of action, this is a *material*, *cognitive*, and *social* interplay—all at once. You are living in the interplay of minds, cities, and societies. Although these three things are complex systems in their own right, the interesting thing is that they end up relating to each other. By working together, minds, cities and societies somehow “merge” into one immensely interactive system.

This article focuses on how humans use information in their environments in order to live and put their actions together in complex systems of interaction. It does so drawing from different traditions in information theory, cognitive studies, complexity theories of cities and social systems theory. It also intends to overcome certain limitations of approaches that deal with these as isolated systems. For instance, we learn from cognitive science about how humans relate to information in their environment. In spite of challenges of empirical demonstration, a number of approaches assert that our minds not only decode information from the environment, but also extend themselves into it. Our cognitive activity is embodied, situated, and shaped by its continuous interaction with the environment [1,2,3,4]. Theories of the extended mind assert a causal flow as the mind uses resources in the environment and vice versa, a two-way interaction [5]. The cognitive system is seen as a network of internal representations (not only of a single person, but several persons) extending into the external environment, as information structures bring changes to “the face of the city” [6,7,8]. We wish to explore other possibilities related to how information is preserved in the built environment, and how it expresses and supports our interactions.

That means exploring how we deal with environmental information in order to perform, and how we make the transition from information to interaction. This transition seems to lie at the heart of a truly systemic problem: how do we put our actions together in a way to create a society? How can individual actions develop into a coherent system of interactions? Or put another way, how can we coordinate individual decisions performed by large numbers of people? We will argue that the way we organize ourselves as societies depends crucially on how we deal particularly with the information encoded in our environment. One thing that minds, cities and societies have in common is information. They depend on it. Minds process information; societies exchange information in order to exist; the built environment contains structures that might be cognized as information. In short, these systems process, share and preserve information. A step further, they seem to relate to each other through information. Following previous approaches, we will argue that this relationship begins with our ability to encode information in the built environment—for instance, producing regularities and differences in urban space. Minds and agency will cognize and enact this environmental information, putting it to use.

Approaching these possibilities requires a number of methodological steps. Once the three-layered conceptual model of information in cities is introduced, we will propose measures of entropy in these different layers, and apply them to selected, emblematic urban cases from different regions of the world, and to simulated scenarios. A measure of environmental information in physical space will allow us to recognize cities with more disordered layouts encoding higher entropy levels. A measure of environmental information in semantic space will suggest that location patterns associated with diverse land use encode higher amounts of information. In turn, a measure of the information enacted by interacting agents will suggest that physical and semantic aspects of space affect coordination in interaction systems.

## 2. Introducing the Information-Interaction System

Shannon and Weaver’s [9] seminal book stimulated most discussions on information and probably is the most important text written on it so far. Interestingly, the way Weaver approached the problem of communication offers a remarkable way to understand how information bridges physical, cognitive, and interaction systems. Essentially, he posed three questions: “(A) How accurately can the symbols of communication be transmitted? (The technical problem.) (B) How precisely do the transmitted symbols convey the desired meaning? (The semantic problem). (C) How effectively does the received meaning affect conduct in the desired way? (The effectiveness problem)” [10] (page 4; cf. [11,12]). We suggest that the relationship between our minds, environment and actions involves similar issues:

1. The *physical* problem: how do we encode and decode information from the environment?
2. The *semantic* problem: how do we make environmental information meaningful?
3. The *pragmatic* problem: how does environmental information affect our actions?

**Figure 1:**
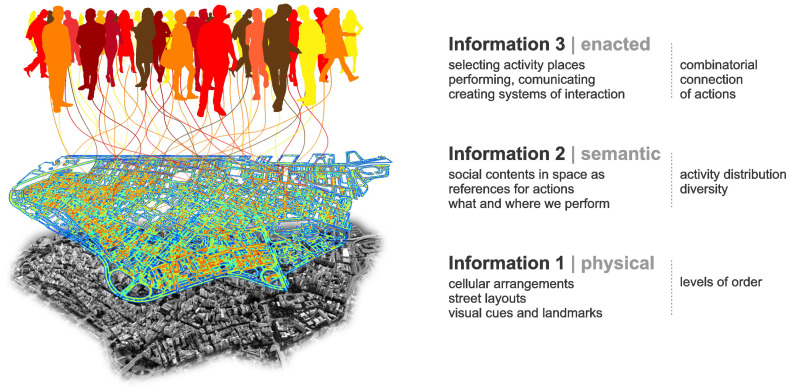
Environmental information (**1**) physical space and (**2**) semantic space, and enacted information (**3**): substantive components and measurable properties. Colours mean different land uses and types of action, i.e., chronically reproduced modalities of work, commerce, leisure and on, usually materialized as activities occurring in buildings and places (see Figure 8).

We will see that information is somehow embedded in tangible spatialities that humans create as their environment. In turn, semantic information is created in the form of meaningful contents, as buildings and places associated with certain activities. We seem to recognize places as settings related to our actions and to a shared idea of what they socially entail. Finally, the pragmatic problem involves how we use information decoded from the environment to guide our actions and create interactions. We propose to handle these forms of information in three overlapping, interacting layers (Figure 1).

Classic spatial theories deal with these forms of information to different extents. For instance, Lynch’s [13] “image of the city” operates mostly at the level of information 1, as it deals with paths and physical cues related to cognition and navigation. Hillier’s [14] space syntax grasps patterns of accessibility in street networks relating to cognition, movement and encounter. Haken and Portugali’s [7,8] synergetic inter-representation networks bridge Shannon and semantic information as basis for actions in the city, but without the systemic aspect of social interaction.

Our theoretical model places the physical form at the bottom as it provides an elementary but fundamental layer of information related to our cognition and navigation in the environment. The *physical layer* includes the arrangement of spatial elements like buildings and streets, and the relations between them. In addition, it is a very stable form of information, changing slowly (cf. [12,15]). In turn, the *semantic layer* has to do with how buildings and places support our actions. Its stability depends on how long actions are performed in those places, and how long their meanings are retained in people’s memories, so it changes more easily. Environmental information 1 and 2 are inseparable, but they are not necessarily intrinsic to each other. Although a building is created to support certain activity, it can be used for different activities in time, sometimes with not need for physical adaptation (e.g., a house becomes a shop or an office). Thus, physical information tends to remain, while semantic information depends on ongoing actions and memories afforded by the building. Finally, the *enacted layer* of information includes the effects that places and their meanings have on agents, bringing possibilities of action [11]. Actions include speech, bodily gestures and the production of signs and objects that carry meaning, and are therefore understandable by other agents, triggering interactions [16,17]. Enacted information is created in the transitions between cognition, action and interaction. It produces and works with environmental information, whenever we use the latter to make individual decisions and communicate.

**Figure 2:**
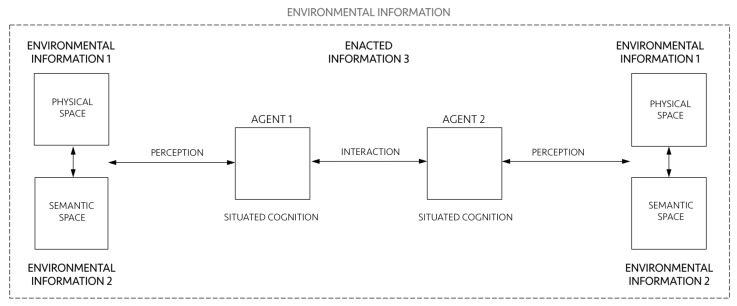
Schematic diagram of a general information-interaction system.

Now let us see what each layer individually is and how they interact as a single system putting minds, cities and societies together (Figure 2). The environment that humans create is composed of tangible and non-tangible structures, an interplay of physical spaces (environmental information 1) and meaningful settings of action (environmental information 2). Environmental information encompasses agents 1 and 2. Agents encode information in and decode information from the environment, and they relate to it through their perception and situated cognition. In turn, cognition is situated because it extends into action and emerges from interactions between agents and their environment. Cognition also emerge from social interactions, as distributed processes in collective operations between agents themselves [18,19]. As we shall see, this extension of cognition into the environment and into how agents coordinate their actions is the very definition of ”enaction” [1], what we call enacted information 3. Enacted information 3 encompasses perception, situated cognition and interactions, and it is deeply related to its environment. Of course, all of these items and relationships involve a vast literature and need proper, detailed definitions. Let us address them following the three-layered conceptual architecture proposed above.

## 3. Environmental Information 1 (Physical Space)

Since Shannon’s [20] pioneering work on the mathematical theory of communication and Wiener’s [21] cybernetics, the notion of information took other disciplines by storm in the 1950s and 1960s [22]. Shannon arrived at a clear description of information through a probabilistic definition of entropy, also explored by physicist Boltzmann [23] before him. For both, entropy is a measure of the uncertainty of a system. The greater the number of potentially transmitted messages (Shannon) or the number of distinct microscopic states of a thermodynamic system (Boltzmann), the higher the corresponding entropy [24]. Shannon’s definition of information was proposed in the context of the problem of transmission of data, noise and channel capacity, from the point of view of engineering ([9], pages 27, 31). He realized that the probability of different arrangements of signs could account for the amount of information embedded in them. Despite the enormous impact of Shannon’s idea in different areas, it has certain implications. Weaver [10] (page 19) himself pointed out that the idea that “greater uncertainty, greater information go hand in hand” seems deeply counterintuitive. Since Boltzmann, entropy is associated with disorder [24,25]. Physical arrangements with higher entropy are characterized by higher levels of randomness, unpredictability or uncertainty. In turn, levels of predictability may be associated with order. Ordered structures contain correlations such as similarities, consistencies and associations that are the “substance” of information [26], like pieces of “coherence above and beyond the bunching and scattering” entities [27] (page 1034). In this sense, information is the pattern of organization of matter and energy [28] (page 10)—like regularities in the arrangement of molecules in a piece of glass, organized parts composing a machine, or the pattern of activity location taking the form of a central business district in a city. A step further, information is a property of recognizable differences and internal correlations in systems that can be decoded by the system itself or by other systems. Information does not require a conscious receiver, but it carries transmissible codes that can guide change in physical processes, behaviour in living systems, and understanding in conscious entities (cf. [29]). It involves intelligibility, but not necessarily “meaning”, as Shannon and Weaver [9] correctly asserted.

The idea that physical things can encode information is not new in theories of cities either. It is at the heart of Lynch’s [13] spatial elements recognized by people, guiding their navigation in the environment, along with memory and representation, even though he did not quite use the term “information”. Rapoport [30] (page 19) explicitly asserted that “physical elements of the environment do encode information that people decode”. Hillier and Hanson [31] thought of non-representational meaning embedded in physical configurations, as patterns guiding way-finding correlated with patterns of co-presence. Haken and Portugali [7,8] have seen information latent in street layouts and built form. Supposing that these theories are right, the fact that information can be encoded in physical structures is very interesting. Information lasts longer when preserved in tangible entities [26]. If physical spaces materialize information, we would have a form of expressing information continuously—as long as these spatialities are out there. We could encode information in the built environment and decode it while living in it. All these forms of information materialized in physical space could be contextual resources useful to guide our actions. Such a property would open extraordinary cognitive and practical possibilities.

In addition, how could humans encode information in the built environment in the first place? At this point, there seems to be no definite answer to this question. Research in spatial information seems to mostly focus on how we decode information from the environment, for instance the role of visual perception, visual variables in navigation, and spatial decision-making (e.g., [32,33]). In turn, empirical work in neuroscience has confirmed that the memory of an environment may be stored as a specific combination of place-cell activities [34]. Neural algorithms integrate information about place, distance and direction, forming a directionally oriented, topographically organized neural map of the spatial environment. ”Grid cells” in the brain are activated whenever the animal’s position coincides with any vertex of a hexagonal regular grid spanning the surface of the environment. Grid cells are critical for vector-based navigation, which can be combined with path-based strategies to support navigation in challenging environments. In addition, the mental map is anchored to external landmarks, but it persists in their absence, suggesting that this grid-like position system may be part of a generalized, path-integration-based map of the spatial environment [35,36,37].

**Figure 3:**
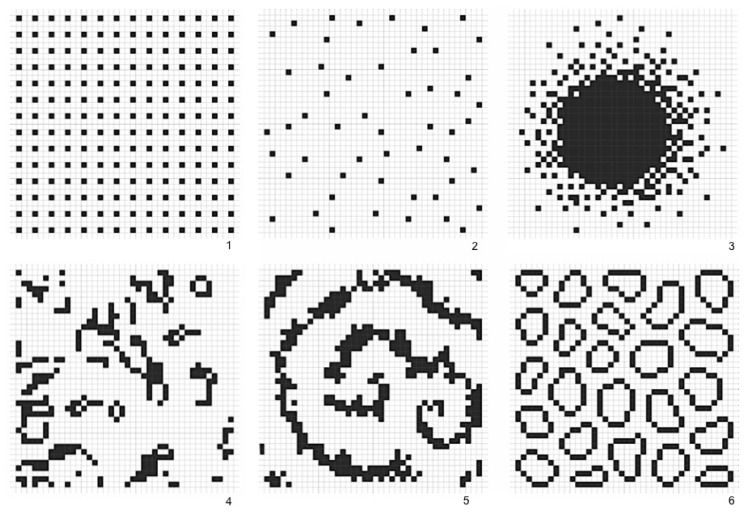
Information in physical space: emblematic arrangements with different levels of order. Case 1 is an extreme example of order, rare in the set of possible arrangements. In cases 2 and 4, cell distributions contain low internal correlations. In case 3, positions follow a patterned distribution, and in case 5 order is visible as a spiraled pattern. The sixth case shows deformed rings, highly unlikely events which bring to mind unplanned urban structures.

In this sense, levels of regularity and predictability in spatial arrangements could be cognitively useful to anchor agents’ internal system of navigation. Agents might capture levels of physical information by recognizing regularity in frequencies of spatial events in the built environment. If that is the case, the greater the variation of elements in the environment, the fewer the regularities that allow inferences about the broader structure. If that is the case, which spatial arrangements contain more physical information? Chess experts show greater memory for chess-typical arrangements of pieces than random arrangements [38]. We suggest that agents create information 1 by engendering levels of order in the deepest constituents of built form, namely cellular aggregations. Imagine a two-dimensional space in the shape of an orthogonal cell grid. Cells occupy positions in this grid, composing different arrangement, like the emblematic cases in Figure 3. These arrangements display different levels of order, apparent in the frequency of distances between cells. An extreme case is the orthogonal arrangement (Figure 3, case 1). Perfectly regular arrangements like this are rare events in the set of possible arrangements, so they seem like drops of order in a sea of disordered states. In most states, cell distribution tends to contain low internal correlations, like cases 2 and 4. In case 3, positions follow a patterned distribution. Some order is also visible in case 5, a spiraled pattern. The sixth case brings deformed rings, like those found in unplanned urban grids. Capable of generalizing contiguity between cells while keeping permeability, the very formation of such rings is a highly unlikely event.

### Measuring Environmental Information 1

**Figure 4:**
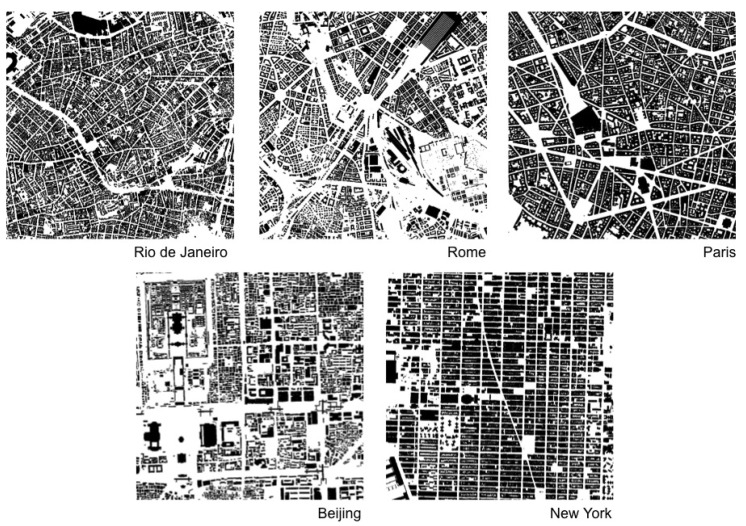
Spatial distributions in real cities (9,000,000 m${}^{2}$ windows, 1000×1000 cells), extracted from Google My Maps. These sections of such emblematic cities will be used to compute Shannon entropy and estimate the degree of disorder in cellular arrangements.

Approaches to visual information have adopted measures of information density and entropy to assess the amount of redundancy and grouping related to cognitive efforts to extract task relevant information [39,40]. We may pursue such possibility measuring levels of predictability in physical arrangements using statistical concepts. A measure of information 1 should be able to grasp regularities and variations in real configurations and different urban situations (Figure 4). For this purpose, we suggest to measure Shannon entropy. As we have seen, high entropy corresponds to high levels of randomness or unpredictability. In contrast, the presence of regularities and patterns in urban structures corresponds to lower entropy. Hypothetically, cities with ordered structures would help agents understand their environment, allowing them to make predictions about areas beyond their fields of visibility. Agents can make inferences, memorize layouts and navigate more easily from one place to another—say, grasping the pattern of blocks and intersections from local streets and inferring that some blocks away they will still have the same pattern (see [13,14,41,42] on legibility and intelligibility in urban structures; References [6,32,33] on pattern recognition and spatial decision making). A measure of spatial entropy can be explored to characterize and classify urban areas and cities from different regions of the world. That is what we attempt to do for a number of emblematic empirical cases. We are certainly aware that a few selected cases do not offer evidence to validate a model. Nevertheless, these empirical analyses are intended as illustrations of the measures at work, displaying their potential utility to capture levels of order and information in spatial arrangements.

We propose to assess Shannon entropy as a proxy to disorder in cellular arrangements extracted from public map repositories of cities such as Google My Maps. Background picture bases were prepared and exported in high resolution, filtering layers and converting entities into solid raster cells. We proceeded to test and analyze trade-offs between resolution, computation and precision in results for distinct scales. We finally chose geographic areas of 9,000,000 m${}^{2}$. Images that underwent a resizing process for $1000^{2}$ cells were converted to a monochrome system through a geographic information system (GIS), and then into a matrix of size 1000×1000 cells with binary numerical values. Then, we employed an approach usually applied for estimating the entropy of sequences of symbols encoded in one-dimensional strings [43]. This approach has been widely used for different type of datasets, from natural languages, speech analysis and behavioural sequences to deoxyribonucleic acid (DNA) and spike emissions in neurons. However, estimating entropy is far from trivial. For datasets corresponding to one-dimensional strings, the most straightforward method consists of defining the block entropy of order *n* by

(1) $$H_{n}=-\sum_{k}p_{n}\left(k\right)\log_{2}\left[p_{n}\left(k\right)\right],$$

where blocks are string segments of size *n*, and the sum runs over all the *k* possible *n*-blocks. Equation (1) corresponds to the Shannon entropy of the probability distribution $p_{n}\left(k\right)$.

**Figure 5:**
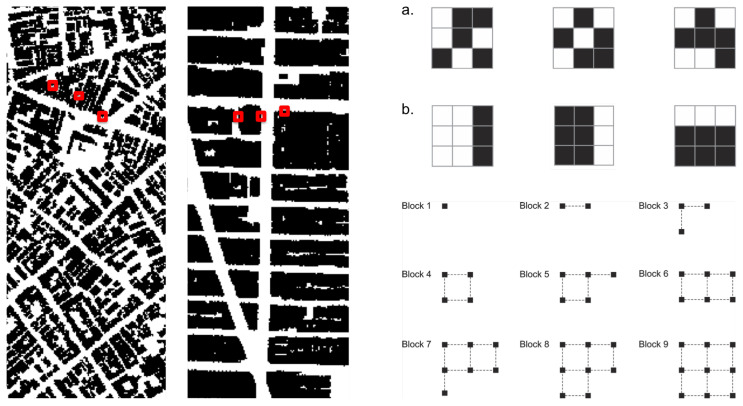
Entropy in configurations of different urban areas: Examples of blocks with nine cells are shown in red for selected areas in Rio and Manhattan, NY (left), and are amplified on the right. Rio shows a great deal of variation of configurations like (**a**). In turn, configurations like (**b**) are frequently found in Manhattan. Essentially, $p_{n}\left(k\right)$ in Equation (1) accounts for the number of times every possible configuration *k* appears in the map for a block of size *n*. A high frequency of certain configurations, like in Manhattan, brings the entropy measure closer to 0, i.e., to higher levels of physical order. This procedure for estimating entropy was applied for blocks with different sizes (i.e., number of cells, below on the right). Here, we show the first nine blocks; other block sizes were generated through this approach. Note that there is no unique natural way to scan a 2D matrix [45]. Different forms of interpolating cells do not seem to influence the estimation of $H_{n}$.

The Shannon entropy of the considered system [43,44] is:

(2) $$h=\lim_{n\to \infty }H_{n+1}-H_{n}=\lim_{n\to \infty }H_{n}/n,$$

which measures the average amount of randomness per symbol that persists after all correlations and constraints are taken into account. This approach can be applied to our problem once we have defined the blocks for a two-dimensional matrix [45]. In this two-dimensional context, the most intuitive idea is to consider a block of size *n* as a square which contains *n* cells. To obtain the sequence of $H_{n}$ also for *n* values that do not correspond to squares, we will consider blocks that interpolate perfect squares, as described in Figure 5.

Relation 2 gives precisely the entropy for a theoretical infinite set of data. In real situations where the dataset is finite, our method estimates the probabilities of distinct arrangements of cells within blocks up to a certain size *n*, counting their frequencies, and then estimates the limit. For example, for $H_{1}$, it is sufficient to have knowledge of the symbol distribution $p_{1}\left(2\right)$, which is approximated by the frequency of 0 and 1 present in the dataset. Note that if our data were a purely random set, *h* would coincide with $H_{1}$, and $p_{1}\left(2\right)$ would give a full account of the spatial configuration. This is obviously not true for urban situations, where strong long-range correlations are present. In this case, estimating entropy is a difficult task, as taking them into account means computing $H_{n}$ for a large *n*. In fact, the estimation of *h* is good when the spatial range of correlations and memory is smaller than the maximum size of the block entropy we are able to compute. This estimation can be rendered difficult because of the exponential increase of the number of distinct cells arrangements in blocks with *n* ($k=2^{n}$). For instance, there are 512 different configurations for blocks with only nine cells. Difficulties in capturing longer correlations lead to the overestimation of *h*. This is the case if sufficient care is used in estimating each $H_{n}$. Otherwise, as strong fluctuations are already present for moderate block lengths *n*, the estimates $H_{n}$ are usually underestimated. These two concurrent effects may jeopardize the estimation of entropy.

**Figure 6:**
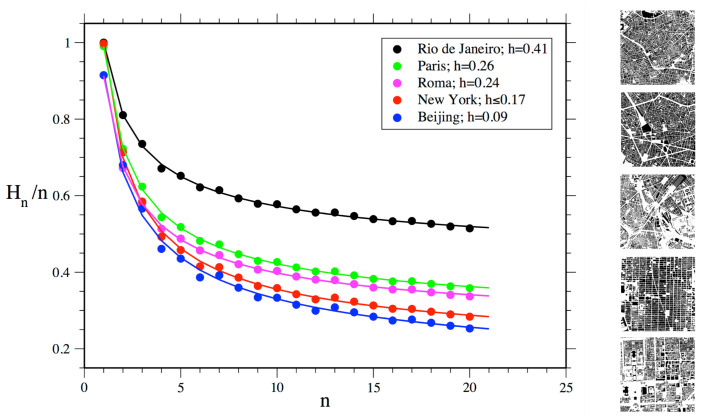
Measuring environmental information 1: estimated values of $H_{n}/n$ for areas in five selected cities. Continuous lines represent the best fitting of our data using the function: $a+b/n^{c}$. The fitted values of *a* give a reasonable extrapolation of the Shannon Entropy *h* of the dataset. Values are reported in the legend. The selected area in Rio de Janeiro shows the highest level of disorder (lower level of physical information) among areas in five selected cities.

In our specific case with just two symbols, the estimation of $H_{n}$ is surely not reasonable when $2^{n}\approx N$, where *N* is the number of elements in our dataset [44]. Thus, in our situation where we work with a matrix with $10^{6}$ cells, this condition is verified for n≈20, which means blocks of squares with a linear length smaller than 5 cells, which corresponds to 15 meters (*m*). It follows that, if a city has internal correlations larger than 15 *m*, the entropy estimation using block entropy will be over-estimated. Results for the estimation of $H_{n}/n$ and the corresponding entropy *h* for areas in five selected cities are shown in Figure 6.

The north area selected in Rio de Janeiro shows the highest level of disorder among selected areas of cities. To a large extent, these results make empirical sense. However, it is less clear why Manhattan would have higher disorder than the area selected in Beijing, given the highly regular grid structure of the former. The reason for this finding can be ascribed to the maximum size of the block of cells used for estimating entropy in our method, which has a linear size much smaller than the actual urban blocks in Manhattan. Limited to the considered cell blocks, our measure is only able to capture sections of Manhattan’s large urban blocks, which consist of very regular alignments of cells on their external borders, but irregular arrangements on their interior. In turn, Beijing’s urban blocks display more variations in shapes and sizes, but are very regular on their external border and interior arrangements.

Finally, this approach allows us to use different sizes of cell blocks for measuring physical information present already at small local scales. Probabilities of empty cells and built cells are similar for blocks with one cell for all analyzed urban areas, suggesting that such cities, selected from different regions and spatial traditions, share a similar proportion of empty and built cells. This surprising finding must be confirmed for larger samples of cities. In turn, block sizes larger than one cell showed stronger differences in block entropy levels between cities, as expected. Differences in values were more pronounced as block sizes increased. These results are consistent with the general expected behaviour for block entropies dependent on the block size for any system. It is interesting to note how physical information can be grasped even at small scales and that disorder is shown to decrease as the size of cell blocks analyzed increases, as larger scales of order and correlation begin to matter.

If we wish to improve the statistical robustness of our analysis by increasing the number of cells in our dataset, we can follow two paths. The first is increasing map size. However, this is limited by the size of cities and by the fact that we need relatively continuous urban areas in order to compare them. Large geographical interruptions like mountains or lakes may interfere with the frequency of blocks configurations, and should be avoided. The second is increasing map resolution. In this case, we must increase the maximum value of *n*. However, the presence of large homogeneous features and the noise produced by finer resolutions increase fluctuations in the estimation of the block entropy, leading to a strong underestimation of entropy. Measures of spatial information can also be introduced through analysis of the fractal dimension of urban structures [46]. Nevertheless, claims of fractality are often made for data that only represent a limited range of scales [47,48]. The characteristic scale of the urban block poses challenges to the idea of self-similarity—for instance, between blocks and the larger spatial structure of Manhattan. A step further, fractal properties are usually found in data characterized by larger scales than the ones we are analyzing in this paper. Therefore, we choose not to connect spatial information with an approach to fractals in this work. Finally, the method we use for estimating Shannon entropy in spatial configurations does not depend on a particular scale. This condition is based on its very formulation and it is proven by the fact that it has been successfully used for estimating entropy in systems that present self-similarity [49].

At this stage, our approach takes account of the physical information latent in the arrangements of cells capturing relations of proximity, but eventually missing some correlations at large distances. However, cellular growth shapes larger structures and paths as fundamental morphological features of cities—a subject explored in other works [7,41,50,51]. In addition, there are other forms of physical information, such as three-dimensional differences between buildings, physical cues and landmarks [52,53]. Forms of estimating the effects of such aspects of physical information will be subject for further work. Levels of regularity in physical space seem useful informational features in cognition and navigation (cf. [7,8,14]). However, are they all that the environment can offer? There might be a limit to the extent of information that physical space can encode, even if ordered to preserve information. Environmental information needs to be more differentiated out if it is to get closer to levels of differentiation found in actions. Thus, how could urban space support more information?

## 4. Environmental Information 2 (Semantic Space)

A number of theories have attempted to expand Shannon’s pioneering ideas on information as messages comprising symbols encoded in well-formed strings of signals into different types of information theory, in a way to deal with the second communication problem pointed out by Weaver [10] (pages 4, 26). That would be the existence of another aspect to information related to the interpretation of transmitted signs, or “how precisely do the transmitted symbols convey the desired meaning”, involving a receiver able to subject the message to a second decoding. Indeed, the specificity of semantic information requires clarification about its place among different types of information theory. In this sense, Floridi [54] identifies three fundamental concepts: (i) information as well-structured data; (ii) information as meaningful well-structured data; and (iii) information as meaningful and truthful well-structured data. Shannon’s theory falls into the first category, since it deals messages comprising uninterpreted symbols. The second category consists of theories of weakly semantic information (TWSI). Finally, theories that place truth as a condition for information fall into the third category, “strongly semantic information” (TSSI). Our approach to (environmental) semantic information falls into the second category: one can encode and decode semantic information from the environment with no resource to statements of truth or untruth. Truth statements do not apply since environmental information does not depend on them to be cognized by agents.

Let us look into these theories a little more closely. The first attempt to deal with the semantic problem of “what symbols symbolise” involving contents or designata of symbols can be found in Carnap and Bar-Hilliel’s [55] (p. 147) formal theory of semantic information. They connected a probabilistic notion of information with the contents of statements in language systems. Later on, Mackay [56] proposed a quantitative theory of qualitative information. Interestingly, Barwise and Perry [57,58] and Dretske [59] have developed such possibilities into a theory: situation semantics. “Situations” are limited parts of the world: events and episodes are situations in time, and scenes are visually perceived situations (Barwise and Perry, 1980). Situation theory is geared to address the way context facilitates and influences the rise and flow of information [60].

Dretske adapted elements of Shannon’s theory to produce a theory of semantic information in which semantic content reduces to the intentional content of information, i.e., physical signs used to transmit encoded symbols produced by a source have intentional content. Furthermore, and also of great interest to our approach, semantic information in Dretske’s sense can also exist as a feature independent of a single, individual mind, and can be quantified. Of course, places may hold different meanings for different people. Like the understanding of words [19], two people probably will not share a same semantic understanding about a place or an environment. A bar may be special for you because you met your wife for the first time there, and I will never know that. The reason for this is that meanings do not lie only in space: they also lie in people’s heads. That means that we could hardly capture the plurality of meanings recognized by a single person in an environment. However, even though the meaning of a place is associated with subjective interpretations, we can access its conventional meaning as a social construction. Meanings are collectively encoded and shared in urban space. I cannot access the special meaning you hold for that bar, but we both realize that that place is a bar, encoded with this social meaning by the kind of recursive actions performed there.

This is known as semantic environmental information. Situation theorists require some presence of information immanent in the environment [54]. The situation supports information: the context contributes to the meaning performed in that context, and vice versa [16,60,61,62]. This is a powerful condition. An environment semanticized by meanings-in-use brings its informational potential to a direct relationship with action. People can recognize the meaning of a place in traces and artefacts left by previous or ongoing actions, and associate these traces with their spatial milieu. Space not only represents the activity, it is also enacted and, as such, laden with meanings. Spaces can “mean” as much as our actions because they are semanticized by our actions. In addition, because space is semanticized by our actions and share the same informational nature, at least in part, it finds a level of differentiation similar to types of actions (see [7,63] on semantic categorization). Semantic information renders the environment endogenous to action.

**Figure 7:**
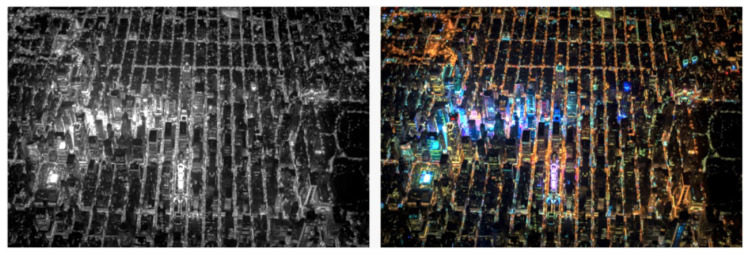
Colours find their own palette of tones, becoming more diverse and contrasting in the passage from grey to colour scales, leading to an enormous increase in combinatorial possibilities. Photo: Vincent Laforet.

The physical environment takes on meaning when spatial elements stand for some social content, and trigger associations with actions. However, what is the informational potential added by semantic meanings encoded in the physical environment? We could think of the difference between environmental information 1 and 2 like the difference between a black and white image and a colour image. In comparison with tones of grey, colours are more diverse and contrasting. New possibilities emerge as each colour finds its own palette of tones, leading to an enormous increase in combinatorial possibilities within such distributions (Figure 7).

Many studies in cognitive science and spatial information theory have asserted associations between physical features and semantic contents in cities. Certain cognitive processes trigger associations with elements of the environment through the incorporation of socially acquired information [64,65]. Information is classified into potentially shared categories [7,60,66]. Non-spatial information can be integrated or associated with spatial information [67], as semantic categorical grouping processes relate to spatial features such as roads to organize point-based locations [63,68,69,70]. Such form of “semantic clustering” can have effects on the memory of spatial locations [71]. The probability of a building or place to evoke a collectively shared mental representation is enhanced by its physical appearance and visual identity, along with its visibility and location in the environment and the social information associated with activities performed there [6,72,73]. In short, in perceiving a building, we perceive not only its form, but also the potential information enfolded in it [7]. We load the built environment with information. Our knowledge of spatial properties and patterns can integrate semantic, visual, and configurational aspects projected into an urban environment as “symbolic off-loading” (cf. [3]). A non-physical thing like meaning may control and change the physical world [74].

However, what does semantic environmental information do for our actions? Semantic associations with places increase the power of the environment to inform us [65,67,71]. For instance, we recall a restaurant more easily if we know that it is located besides a mall or another restaurant. Locational patterns like that work cognitively: activities from a same industry will frequently be located near each other whether they compete or establish forms of complementarity by similarity [75], either way attracting more potential attention than if they were dispersed and isolated. Importantly, different forms of specialization frequently overlap within a same area [76,77,78]. A heightened amount of semantic information can be found both in the concentration of diverse activities and the specialization of areas [79]. If an area has more dense and diverse activities, our chances of finding something we need an increase, and we can guess that from visual and semantic cues. In other words, encoding the physical world with semantically structured models brings advantages. It enables us to build *inferences*. This property is crucial for our actions. Diverse and concentrated activities in urban areas trigger more possibilities [52]. People can build instructions about such possibilities in the environment in an indexical form [80]. Agents can find cognitive and practical synergy manifested in the form of proximity, categorical grouping and clustering [81]. Local adjacencies between different activities may create interesting complementarities from the point of view of agents in their efforts to perform and coordinate their actions.

### Measuring Environmental Information 2

**Figure 8:**
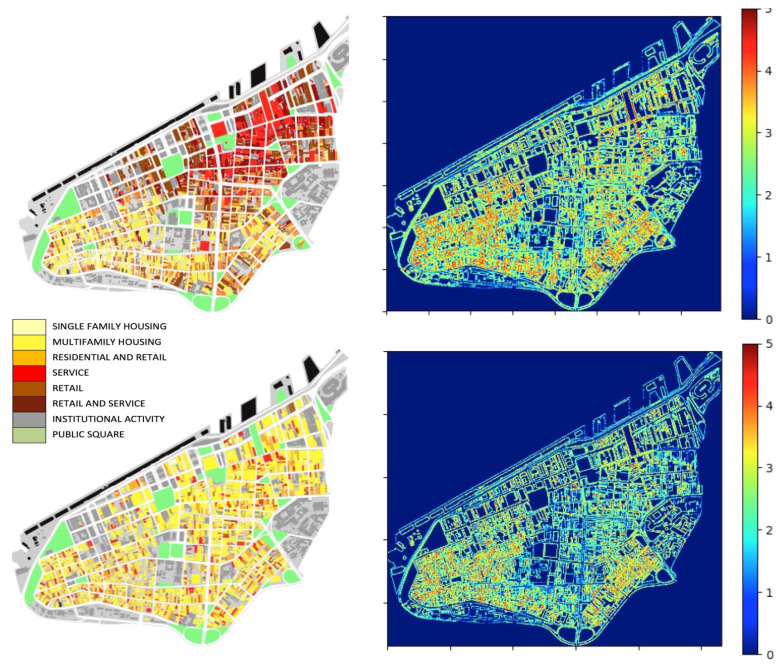
Semantic maps: real distribution (**top**) and fictitious distribution (**bottom**) of land uses in Porto Alegre’s central business district (CBD), Brazil. Corresponding $H_{j}^{m}$ values are shown on the right. Values shown on the right (from blue to red) are calculated with Equation 3. Values are calculated for m=10, with the cell *j* corresponding to a pixel of the maps on the left. The calculation is run over a gray-scale copy of the maps. Source: Authors based on [75].

Semantic similarity has been proposed as a measure to determine the difference between feature type definitions [81,82]. We argue that a key point is to measure the diversity of social contents in buildings and places.We propose to deal with such shared meanings through semantic maps [83]. Semantic maps can represent any form of social content in urban space, including kinds of interaction in activity places. They do not necessarily capture individual interpretations and private memories of places, but they can capture their social meanings. These meanings are part of the knowledge of a social world and its environment, and should be enough to support our understanding of the collective role of places, i.e., their role in supporting actions and interactions. Like any kind of social and environmental knowledge, it must be built heuristically in situation-based behaviour in our daily interactions and context [6,18,84,85]. For simplicity, we choose classic categories in urban studies, namely land uses varying from public squares to residential buildings, and apply them in the empirical analysis of two archetypal urban areas (Figure 8, left). Land uses are variables usually employed in analytical procedures in urban modelling.

Semantic information can be measured as levels of diversity found in land use distributions, analyzing local adjacencies between neighbour cells. In principle, we could do so by measuring entropy using the same procedure used for information 1. Unfortunately, the fact that now we should analyze a system characterized by more than two numerical values is an obstacle for a reasonable estimation of frequencies, which limits the effective possibility of estimating the *h* values. For this reason, we suggest a different measure. For each cell *j*, we define a block of cells correspondent to a square of linear size *m*, centered in *j*. Inside this square, we measure the land use distribution, which is approximated by the frequency $f^{m}\left(z\right)$ of the different *z* values (types of land use) present in the block. For instance, in both cases in Figure 8, there are z=8 types of land use. Then, we estimate the value of $H_{j}^{m}$ for the *j*-th position, given a neighbour cell of size *m*:

(3) $$H_{j}^{m}=-\sum_{z}f^{m}\left(z\right)log_{2}\left[f^{m}\left(z\right)\right].$$

Note that even though this measure is partly analogous with the previous entropy estimation, it is indeed different. We are simply measuring an entropy-based scalar index for characterizing the local distribution of different land uses in a given neighbourhood of *j*. This process can be run for all cells present in the matrix, and it is analogous to some algorithms used in image processing. A graphic analysis representing all the estimated $H_{j}^{m}$ values can be produced. Applying this approach to these two archetypal cases, we obtain results seen in Figure 8 (right).

The graphic analysis identifies cells of high diversity in both maps, corresponding to local interfaces between land uses, a form of information potentially attractive to agents. However, the real distribution contains a higher number of spots of cells of diverse semantic information than the fictitious one. This distinction suggests the potential usefulness of this method to capture levels of information 2 in different distributions of social contents in cities. Nevertheless, let us see how these two forms of environmental information can be part of the interaction.

## 5. Information 3 (Enacted)

Reminding Weaver’s [10] third problem of communication (“How effectively does the received meaning affect conduct”), we finally reach the problem of how agents use environmental information 1 and 2 to interact. We called this “enacted information” or information 3. The term “enaction” can be found in a number of approaches, from Bruner’s (1966) description of knowledge as acquired and manifested through action to Varela’s [1] “paradigm of enaction”. Our use of the term shares aspects especially with the latter. The approach relates to emerging paradigms based on “embodiment”, “situatedness”, and the relevance of cognition for action [3,86]. Enaction is “embodied” in the sense that cognition depends upon the experience of a body with sensorimotor capacities “situated” in a more encompassing biological, psychological and cultural context [1]. A step further, agents enact their cognitive domain by actively and asymmetrically regulating the conditions of their exchange with their environment. Enactive approaches evolved in opposition to computational cognitivism, and reject the traditional pole of seeing the mind as only responding to environmental stimuli [19]. Instead, enactive approaches focus on how sensory inputs from the environment guide actions, and actions modify sensory returns and the environment itself, in perception-action loops. Interaction with the physical and social environment would make measurable differences in cognition and vice versa [1].

Embodied interaction is also emblematic in enaction research. It is seen as “mutual participatory sense-making” involving the emergence of roles, values, dispositions to act, and meanings [1]. Meaning belongs to the relational domain established between the internal dynamics of the agent and elements in the environment. It is inseparable from the context-dependent, embodied activity, without being beyond the reach of scientific understanding. Agents “cast a web of significance on their world”: exchanges with the world are inherently significant for the agent. In turn, meaning is derived from information processing. Through meaning and interaction, agents extend beyond the strict confines of their bodies, into the socio-linguistic register [1].

At this point, we reach a key problem in enactive approaches: *how coordination emerges between agents*. The emission and reception of signals in language gives rise to the modulation and coordination of actions. Interestingly, there is communication only if signal-mediated interactions result in coordination of actions [87]. The semantic layer of information endows the enactive layer with a fine-grained medium to convey meanings in communication, and vice versa [11]. This inherent connection is consistent with previous approaches we have seen, namely the idea that agents rely on environmental information to perform.

However, what exactly is the practical role of environmental information in this coordination of actions? Does it have to do with interaction and the creation of systems of interaction? We have seen that buildings and places mediate interactions. A physically and semantically structured space becomes *the referential frame that provides a form of organization to the field where interactions are performed*. Places become nodes in the unfolding connections between actions. These connections end up amounting to networks of interactions between places, and between people and what they do. Let us see how this happens. Think of a place you might go in your daily life—say, in a busy street a couple of corners away from your home. You will not go there by sheer luck or fate. The physical and semantic environment has informed you that that the place could exist, and that it was a possibility for your action, a means to join a social situation and take part in the huge system of interactions that structures the world around you. The physical and semantic environment plays an active part in how you and other agents there managed to reach some agreement or do something together, coordinating your collective actions. In addition, that situation can be extrapolated to other situations. What happens there tends to have ripple effects, with actions and their outputs connecting to other actions and places, merging into a system of interactions that unfolds in larger scales through different media, including the built environment of cities. In short, *environmental information becomes a means of social organization*—or, in information theory terms, a means of reducing (social) entropy.

**Figure 9:**
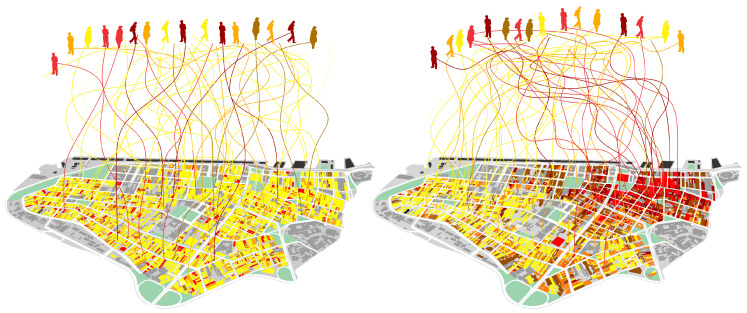
Diagram of agents converging in a fictitious, nearly random distribution (**left**), and in a real, patterned one (**right**).

Now, given the enormous number of possibilities of interactions in a city [51], how precisely can the environment inform us and help us select places and actualize interactions? How much do different physical patterns (information 1) affect this combinatorial process? Would different levels of diversity in activities (information 2) have different effects on levels of entropy in people’s actions? We propose to assess probabilities of combinations of actions in different spatial scenarios, such as those in Figure 9.

One way to do this is quantifying the amount of environmental information available for agents in their decisions. We tested that idea through an “enaction model” able to assess information 3 proposed in a companion article (Netto et al. [88]). Like situation semantics, our model considers agents, types of action, spatial and temporal locations, situations (represented by activity places) and parameters able to capture aspects of the agent’s cognitive behaviour, namely the ability to track and recognize social situations, make decisions based on their individual orientations, and change their own actions and the semantic environment. Our agent-based model (ABM) based on a city as a linear structure is able to represent the minimal sufficient aspects of environmental information 1 (physical distance between positions representing places) and information 2 (semantic differences in the information contents of cells). The linear form of this city allows continuous movement across a sequence of locations, eliminating centrality factors while taking into account periodic boundary conditions in order to reduce border effects, eliminating the role of topology while isolating the problem of physical distance [89]. Our hypothesis is that as (a) physical proximity latent in environmental information 1 tends to increase interactions between agents [90,91,92], its association with (b) environmental information 2 created by diversity in locational patterns increases the potential for interactions and the coordination of actions, leading to reductions in the entropy of action in enacted information 3.

In each time-step, agents select and visit a specific cell within the city. The decision on which action to perform next may be influenced by three different conditions: namely, (i) latent orientation, the tendency of a single agent to act around a particular type of action, based on long-term memories of how to act, shaping (along with short-term memory) current actions. They are initially randomly distributed, and this condition remains over time. (ii) Current action performed by an agent as she selects an activity place in order to perform a new action; this means an influence of the current action, while allowing gradual changes of orientation in time influenced by other agents and activities. (iii) Activity places where agents perform specific types of action. Agents select activity places closer to their latent orientations and current actions, while being influenced by those activities. Visiting agents also influence places, but places change at a slower rate. Agents co-evolve with their semantic environment.

Let us see how action orientations change in time *t*. Action orientation for agent *i*—from a population of *N* agents—is represented by $\sigma_{i}\left(t\right)$; while the type of activity in a place located at the position *x*, is represented by $\sigma_{x}\left(t\right)$. We quantify and update the action orientations of agents weighting three variables: (i) latent orientation $\sigma_{i}^{in}$, which does not change in time; (ii) current action $\sigma_{i}\left(t\right)$; and(iii) activity place $\sigma_{x}\left(t\right)$. Orientations will be updated according to the rule:

(4) $$\sigma_{i}\left(t+1\right)=\alpha \sigma_{i}^{in}+\beta \sigma_{i}\left(t\right)+\gamma \sigma_{x}\left(t\right),$$

where α, β and γ are parameters of the model. The activity in places will be updated according to the rule:

(5) $$\sigma_{x}\left(t+1\right)=\sigma_{x}\left(t\right)+\theta \sum_{i\in x}\sigma_{i}\left(t\right),$$

where the sum runs for all agents in place *x* at time *t*. Parameter θ is a sufficiently small quantity to grant that changes in activity places will be slower than changes in the actions of agents. In short, places are less influenced by agents than the other way around. This means that, at every time-step, a place would have its activity closer to the average of orientations of its visitors. Agent *i* chooses a place to visit at time *t* based on the similarity (i.e., numeric proximity) of her action orientation and the activity ongoing in a place. A possibility is to consider that agent *i* chooses to visit a place that minimizes the function $E_{i}\left(x,t\right)=\left|\sigma_{i}\left(t\right)-\sigma_{x}\left(t\right)\right|$. Our ABM simulated two scenarios: a first one where physical space and distances do not matter, or a materially inactive space; and a second one where physical space matters, or a materially active space. In the first scenario, the agent chooses a place to visit based only on the similarity between her action orientation and the activity located in space, i.e., minimizing $E_{i}\left(x,t\right)=\left|\sigma_{i}\left(t\right)-\sigma_{x}\left(t\right)\right|$. In the second scenario, the agent will take into account not only similarity, but the physical distance to the activity located at *x*, giving preference to closer places of a similar type. For example, in this scenario, an agent will choose the place that minimizes the function $E_{i}\left(x,t\right)=\left|\left(x_{i}-x\right)\cdot \left(\sigma_{i}\left(t\right)-\sigma_{x}\left(t\right)\right)\right|$. Summing up, an agent *i* selects at time *t* a particular activity place located at *x* that minimizes the function $E_{i}\left(x,t\right)$ (see [88]).

Enacted information is measured as a function of entropy: consider N(σ,t) the number of agents with an action orientation σ at the time *t*. The total population is $N=\sum_{\sigma }N\left(\sigma ,t\right)$). We can compute the frequency (or density) ρ(σ,t) of this orientation within a population with:

(6) $$\rho \left(\sigma ,t\right)=\frac{1}{N}N\left(\sigma ,t\right).$$

The entropy level for any distribution of orientations is:

(7) $$S\left(t\right)=-\sum_{\sigma }\rho \left(\sigma ,t\right)\ln\left(\rho (\sigma ,t)\right).$$

**Figure 10:**
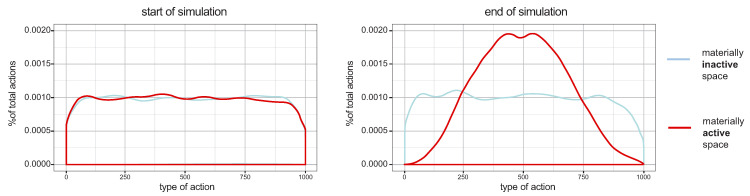
Entropy in interactions: histograms of probability distribution of action types show high levels of entropy at the start of simulations (**left**) in two kinds of scenarios: where distance between activity places is not considered (blue line), and where distance is considered (red line). At the end of simulations (**right**), scenarios where space is materially active show convergence around certain types of action, indicating a reduction of entropy in action coordination.

This describes how uneven the probability is of finding different action orientations. Higher values mean that different actions have almost the same probability to happen, while lower values indicate a system with clear action trends. The reduction of entropy implies that the probability of certain actions increases, that is, actions grow in similarity. In the limit, as entropy falls to zero, all agents in the system would reach the same action. Differences in the weight of factors over the next action may lead to quite different levels of social entropy, in interfaces of simplified systems of action, information, and space. We tested two kinds of scenarios: one where proximity between cells latent in physical information does not matter to agents in their decisions (blue line) and one where it matters (red line). Figure 10 shows the probability distribution of actors performing different action types at the start (left) and at the end (right) of simulations, The scenario where proximity between activity cells matters increases dramatically the coordination of action. Results are averaged for 30 runs for each of 125 combinations of parameters.

Our results show structure in the relation between selection factors and the reduction of action entropy. Informational contents in urban space play a key role in the reduction of action entropy (red lines in Figure 10). Urban space materializes gradients of difference in potential interactions, from less to more recognizable, costly or likely. Placing simple criteria like proximity as an aspect of physical information in selecting activities, our model shows that space becomes a means for producing differences in the probabilities of interaction, increasing chances of certain selections. Environmental information “contaminates” agents: they are likely to align their actions through the informational content of places. This general reduction of entropy implies that the probability of certain interactions increases. In practical terms, this means more alignments between agents. As an information environment, *the physical and semantic environment creates differences in the probabilities of interaction*, helping to solve the combinatorial problem of connecting actions into a system.

## 6. From Information to Interaction: Concluding Remarks

In this paper, we attempted to understand how humans use information in their environment in order to live together and create systems of interaction. We did so (a) proposing a three layered model of information in cities; (b) developing measures for each of the layers and testing them in selected empirical cases from different regions; (c) exploring an ABM to simulate how aspects of environmental information affect social entropy. Previous works dealt only with aspects of these relations, like spatial paths and physical cues related to cognition and navigation [13], patterns in street networks related to cognition and encounter [14] and synergetic networks as basis for actions in the city [7,8], but without the systemic aspect of social interaction. In turn, our model of information enacted by agents (information 3) shows that aspects of environmental information in physical and semantic space contribute to increases in coordination in interaction systems. To the best of our knowledge, no other work integrates cognition, environment and social systems as comprehensively.

More specifically, inspired by Weaver’s [10] levels of communication problems, we addressed the relation of minds, cities and societies through three questions: (i) How do we encode and decode information from the physical environment? (ii) How do we make environmental information meaningful? (iii) How do we use environmental information to coordinate actions? Methodologically, this implied three problems: measuring information 1 in physical space; measuring information 2 in the distribution of semantic contents in urban space; and modelling how agents deal with environmental information 1 and 2 in order to coordinate actions in information 3. Developing a framework to answer those questions, we explored different strands of information theory and cognitive studies, from Shannon’s theory of well-structured data to Dretske’s situation semantics and Varela’s concept of enaction, and did so in the following directions:

1. We measured environmental information 1 in cellular arrangements and assessed order, assumed to guide navigation, through Shannon entropy.
2. We measured environmental information 2 as a function of semantic diversity in local relations between urban activities or land uses. Information 2 is highly differentiated, serving as a resource of great combinatorial power in the process of selection of places and actions to be performed.
3. We modeled the influence of the environment in enacted information 3, simulated as action types in an ABM. Proximity in physical configurations in information 1 and semantic contents in information 2 were seen to increase the convergence of action types. Contrary to isolated systems where order inexorably dissipates in time, the entropy of interaction decreases as the system of agents is open to and co-evolves with its physical and semantic environment. Aspects of information 1 and 2 find key roles in solving the combinatorial problem of action coordination.

A number of procedures are underway for further developing this approach. First, we intend to expand the approach to information 1 looking into broader spatial structures in cities—assessing measures of visibility [14] and the entropy of angular variation in street networks [42]. We also wish to go beyond regularity and entropy, and introduce more sophisticated measures of statistical complexity. Second, we hope to expand the approach to information 2 beyond diversity, looking into the effects of specialization and concentration of social activities, especially those with power to attract agents looking for associations based on functional complementarity. Another issue is the integration of physical and semantic environmental information. Recent approaches have paid attention to this relationship in different ways [8,81]. Information 1 seems associated with orientation and navigation, while information 2 seems closer to the perception and selection of performance opportunities. They also change according to different temporalities. These forms of environmental information are deeply associated but cannot be reduced to one another. Relations between configuration, physical cues and semantic references remain a key area for further study.

We also expect to develop a database of environmental information for a large number of cities from different regions of the world, which will allow us to systematically assess them as informational resources, an aspect of urban, social and economic performance. We hope to see whether different cities carrying different levels of environmental information could affect how inhabitants perform and coordinate their actions. Our hypothesis is that certain environments would ease cognition, navigation and efforts of interaction.Finally, these three forms of information, particularly information 3, are hard to assess empirically. In order to validate this model, we hope to explore the heuristics of how people actually perceive and enact information in the built environment through cognitive experiments performed in Virtual Geographic Environments (VGE) [84,93].

Interaction systems require a high capacity to access and recombine information into on-going and future connections. They are ‘information-hungry’. This dependence requires different types of information. Environmental information seems to play a part in the continuous creation of interactions. The fact that cities preserve social information in durable space and in soft semantic structures would help order grow. Only by means of a structure that restricts the quasi-endless combinatorial possibilities of interaction, this system can acquire sufficient internal guidance to make its own reproduction possible. By distributing sufficiently recognizable differences in the probability of interactions, cities ease the local reproduction of a society. The city helps us convert information into interaction. In fact, the interfaces of cognition, environment and action reveal a deep connection. Self-organizing in themselves, they also seem co-dependent—and more. They seem to shape each other andemerge as an integrated system in its own right. This integration of minds, cities and societies happens through information. Information is the bridge.

References
